# Supplementary material for: Understanding multimorbidity trajectories in Scotland using sequence analysis
Source: Sci Rep. 2022 Oct 1;12:16485. doi: 10.1038/s41598-022-20546-4 (PMC9526700; doi:10.1038/s41598-022-20546-4)
Supplement: Supplementary file 1 — Supplementary Information 1. [file 41598_2022_20546_MOESM1_ESM.docx]

**Additional file 1. Cluster quality measures for different specifications of the indel value**

**C**luster quality measures allow the identification of the best clustering solution. A range of measures are available in R using the wcKMedRange function from the WeightedCluster library: PBC - Point Biserial Correlation, HG - Hubert's Gamma, HGSD - Hubert's Somers D, ASW - the average value of the silhouette, ASWw - the average value of the silhouette weighted, CH - Calinski Harabasz Index, CHsq - CH squared, R2 – Pseudo R2, R2sq – R2 squared, HC - Hubert's C.

For interpretation, an ASW measure close to 1 means the corresponding n-clusters solution is well clustered. A HC close to 0 means good clustering. For other measures (PBC, HG, HGSD, CH, CHsq, R2, R2sq), higher values mean better clustering.

The following practical guide developed by Matthias Studer provides further information (with code examples and reference publications) about these cluster quality measures:

Studer, Matthias. "WeightedCluster library manual: A practical guide to creating typologies of trajectories in the social sciences with R." (2013).

To decide on the best number of clusters and its related partition, we follow some principles, aligning with Studer’s guide. First, we recognise that some the cluster quality measures provided by the wcKMedRange function from the WeightedCluster library are less useful than some others. For example, the pseudo R2 does not penalize for complexity. This measure and its squared version keep increasing as the number of clusters increases. More generally, a measure that always increases or always decreases as the number of clusters increases (e.g. R2, HG) is not necessarily useful to discriminate the best cluster solution. Therefore, these measures are not deemed appropriate to compare partitions with different number of groups. We do not use R2, R2sq and HG to judge on the best number of clusters to choose from. Furthermore, a solution with too many clusters is not very practical for further meaningful interpretation so we have chosen a solution with ten or less clusters. Finally, the choice of number of clusters remains a judgment call and using cluster quality measures should be taken as a guide for decision. We will choose the solution that seemed to gather most of the best results from a range of quality measures.

See below the cluster quality measures obtained for the sensitivity analyses using varied Indel values: 0.5, 1, 1.5 and 5.

1. Indel value of 0.5

PBC HGSD ASW ASWw CH CHsq HC

cluster2 0.22 0.26 0.19 0.19 579.61 927.50 0.32

cluster2 0.30 0.35 0.20 0.20 857.88 1415.50 0.29

cluster3 0.39 0.42 0.21 0.21 839.68 1441.17 0.30

cluster4 0.50 0.59 0.25 0.25 854.63 1587.74 0.25

cluster5 0.52 0.63 0.28 0.28 867.15 1706.25 0.23

cluster6 0.59 0.76 0.29 0.29 891.64 1925.04 0.17

cluster7 0.63 0.85 0.32 0.32 876.36 2069.86 0.12

cluster8 0.64 0.87 0.32 0.32 821.35 2033.51 0.11

cluster9 0.61 0.87 0.29 0.29 781.16 1966.13 0.12

cluster10 0.57 0.86 0.26 0.26 749.67 1900.78 0.13

cluster11 0.55 0.87 0.25 0.26 718.48 1835.90 0.13

cluster12 0.54 0.86 0.25 0.25 691.64 1757.47 0.13

The cluster quality measures above point to a good clustering for a 7 or 8-clusters solution.

1. Indel value of 1

PBC HGSD ASW ASWw CH CHsq HC

cluster2 0.28 0.30 0.18 0.18 708.33 1208.42 0.29

cluster3 0.44 0.51 0.21 0.21 701.68 1227.04 0.28

cluster4 0.46 0.53 0.21 0.21 726.84 1296.62 0.29

cluster5 0.54 0.68 0.24 0.24 753.78 1461.07 0.23

cluster6 0.55 0.71 0.26 0.26 769.04 1550.72 0.22

cluster7 0.58 0.75 0.27 0.27 725.08 1534.57 0.20

cluster8 0.56 0.77 0.25 0.25 688.42 1476.17 0.19

cluster9 0.57 0.81 0.25 0.25 656.75 1436.61 0.17

cluster10 0.57 0.83 0.26 0.26 635.28 1458.59 0.16

cluster11 0.55 0.82 0.25 0.25 621.47 1433.91 0.16

cluster12 0.55 0.83 0.25 0.25 608.45 1429.10 0.16

The cluster quality measures above point to a good clustering for a 6 or 7-clusters solution.

1. Indel value of 1.5

PBC HGSD ASW ASWw CH CHsq HC

cluster2 0.20 0.18 0.15 0.15 715.86 1160.98 0.34

cluster3 0.40 0.44 0.19 0.19 707.58 1212.64 0.31

cluster4 0.42 0.46 0.21 0.21 718.62 1252.24 0.31

cluster5 0.54 0.66 0.24 0.24 728.95 1390.58 0.23

cluster6 0.59 0.75 0.26 0.26 726.03 1472.11 0.20

cluster7 0.60 0.77 0.26 0.27 693.23 1467.16 0.19

cluster8 0.57 0.76 0.25 0.25 667.18 1430.63 0.20

cluster9 0.59 0.82 0.25 0.25 645.86 1443.59 0.17

cluster10 0.58 0.83 0.26 0.26 631.20 1451.06 0.16

cluster11 0.56 0.84 0.26 0.26 619.54 1450.08 0.16

cluster12 0.57 0.87 0.27 0.27 604.00 1497.33 0.14

The cluster quality measures above point to a good clustering for a 7-clusters solution.

1. Indel value of 5

PBC HGSD ASW ASWw CH CHsq HC

cluster2 0.20 0.18 0.15 0.15 716.10 1161.31 0.34

cluster3 0.40 0.44 0.19 0.19 707.81 1213.05 0.31

cluster4 0.42 0.46 0.21 0.21 718.80 1252.60 0.31

cluster5 0.54 0.66 0.24 0.24 729.19 1391.17 0.23

cluster6 0.59 0.75 0.26 0.26 726.29 1472.87 0.20

cluster7 0.60 0.77 0.26 0.27 693.47 1467.94 0.19

cluster8 0.57 0.76 0.25 0.25 667.41 1431.37 0.20

cluster9 0.59 0.82 0.25 0.25 645.01 1439.90 0.17

cluster10 0.58 0.83 0.26 0.26 630.38 1447.20 0.16

cluster11 0.56 0.84 0.26 0.26 618.60 1445.43 0.16

cluster12 0.57 0.87 0.27 0.27 602.56 1489.51 0.14

The cluster quality measures above provide very similar results to that of the analysis with an indel of 1.5 and point to a good clustering for a 7-clusters solution.
